# Hydrogen Sulfide Inhibits Autophagic Neuronal Cell Death by Reducing Oxidative Stress in Spinal Cord Ischemia Reperfusion Injury

**DOI:** 10.1155/2017/8640284

**Published:** 2017-06-08

**Authors:** Lei Xie, Sifei Yu, Kai Yang, Changwei Li, Yu Liang

**Affiliations:** ^1^Department of Orthopedics, Ruijin Hospital, Shanghai Jiao Tong University School of Medicine, 197 Ruijin 2nd Road, Shanghai 200025, China; ^2^Shanghai Key Laboratory for Prevention and Treatment of Bone and Joint Diseases with Integrated Chinese-Western Medicine, Shanghai Institute of Traumatology and Orthopedics, Ruijin Hospital, Shanghai Jiao Tong University School of Medicine, 197 Ruijin 2nd Road, Shanghai 200025, China

## Abstract

Autophagy is upregulated in spinal cord ischemia reperfusion (SCIR) injury; however, its expression mechanism is largely unknown; moreover, whether autophagy plays a neuroprotective or neurodegenerative role in SCIR injury remains controversial. To explore these issues, we created an SCIR injury rat model via aortic arch occlusion. Compared with normal controls, autophagic cell death was upregulated in neurons after SCIR injury. We found that autophagy promoted neuronal cell death during SCIR, shown by a significant number of terminal deoxynucleotidyl transferase-mediated dUTP nick end labeling- (TUNEL-) positive cells colabeled with the autophagy marker microtubule-associated protein 1 light chain 3, while the autophagy inhibitor 3-methyladenine reduced the number of TUNEL-positive cells and restored neurological and motor function. Additionally, we showed that oxidative stress was the main trigger of autophagic neuronal cell death after SCIR injury and N-acetylcysteine inhibited autophagic cell death and restored neurological and motor function in SCIR injury. Finally, we found that hydrogen sulfide (H_2_S) inhibited autophagic cell death significantly by reducing oxidative stress in SCIR injury via the AKT-the mammalian target of rapamycin (mTOR) pathway. These findings reveal that oxidative stress induces autophagic cell death and that H_2_S plays a neuroprotective role by reducing oxidative stress in SCIR.

## 1. Introduction

Spinal cord ischemia reperfusion (SCIR) injury is a severe complication of thoracoabdominal aortic surgery and leads to paraplegia in approximately 40% of patients [1]. Despite considerable attempts to improve SCIR injury, the incidence of paraplegia remains high [2, 3].

The pathophysiologic changes underlying ischemia reperfusion (I/R) injury involve necrosis and apoptosis [4]. In the central nervous system, I/R injury triggers a complex series of pathophysiological events leading to cell death and organ damage by the amplification of various pathways activated by ischemia [1, 5]. Recently, autophagy has attracted scientists' attention worldwide, as a new mechanism of cell death [6]. It has been reported that spinal cord injury (SCI) upregulated the expressions of microtubule-associated protein 1 light chain 3 (LC3, a marker of autophagy) and beclin-1 in a hemisection mouse model [7]. Autophagy is an intracellular bulk degradation process that involves the degrading and recycling of cytosolic, long-lived proteins and organelles [8]. Usually, autophagy occurs at basal levels, but it can be further induced by ischemia, hypoxia, or nutrient depletion [8, 9]. In a certain extent, autophagy ensures the stabilization of the cell and thereby cell survival, by recycling new cell components [6, 10, 11]. But beyond this range, autophagy leads to the excessive accumulation of autophagosomes and eventually cell death [6]. In some studies, autophagy has been shown to contribute to cytoprotection in neonatal hypoxia-ischemia-induced traumatic brain injury [7]; however, other studies have shown that autophagy can cause cell death, termed autophagic or “type 2” cell death [12, 13]. The function of autophagy may thus be two-fold. However, in spinal cord I/R injury, its role is still controversial and it remains to be elucidated whether autophagy is required for cell survival or whether it plays a detrimental role [8, 14, 15].

The mechanisms of cell death that have been observed during I/R injury are complicated. It has been shown that free oxygen radical-induced cell damage plays a pivotal role in the pathogenesis of SCIR injury [5]. Reduction in spinal cord blood flow (ischemia) after aortic or spinal surgery causes hypoxia in the spinal cord and increases levels of lactic acid, hypoxanthine, and lipid peroxide. After the restoration of the blood flow (reperfusion), oxygen is needed for the production of uric acid. During this period, free radicals are produced and react with cellular lipids and mitochondrial membranes to produce lipid peroxides, which cause cell death and organ damage. This process is called “reperfusion injury” [5, 16]. Furthermore, oxidative stress is also thought to play a role in the induction of autophagy and induces neuronal damage in spinal cord I/R injury. Therefore, pharmacological therapies targeting oxidative stress may be critical for limiting the damage caused by SCIR injury.

Hydrogen sulfide (H_2_S) is an endogenously generated gaseous signaling molecule produced from L-cysteine in the myocardium, fibroblasts, and blood vessels by the enzymes cystathionine *β*-synthase and cystathionine *γ*-lyase in the cardiovascular system [4]. As a gaseous signaling molecule, H_2_S is able to freely diffuse across cell membranes in a receptor-independent manner and activate various cellular targets. This distinct ability makes H_2_S an attractive pharmacological agent for the treatment of cardiovascular disease [17]. Myocardial I/R injury is a common problem in clinical practice and may have serious consequences. The exact pathophysiological mechanism underlying myocardial I/R injury is complicated and not yet fully understood. However, there is considerable evidence suggesting that the production of reactive oxidative species (ROS), and subsequent ROS-related cellular damage, is an initial cause of injury to the myocardium following I/R injury [18]. H_2_S has been implicated in alleviating the pathological processes induced by myocardium I/R injury by reducing levels of ROS [19–21]. In addition, it has been shown that H_2_S attenuates myocardial hypoxia-reoxygenation injury by inhibiting autophagy via the mammalian target of rapamycin (mTOR) activation and protects the myocardium against I/R injury by inhibiting apoptosis via a mechanism that involves phosphoinosmde-3-kinase (PI3K)/AKT [22]. Since ROS-induced cell damage also plays a pivotal role in the pathogenesis of SCIR injury [5], here, we sought to investigate whether H_2_S can attenuate SCIR injury by reducing oxidative stress.

Numerous studies have reported that autophagy activation is elevated after SCIR, but the underlying mechanisms are largely unknown. Moreover, whether autophagy exerts a neuroprotective or neurodestructive role in SCIR injury remains controversial. We set out to explore these issues using an SCIR rat model created via aortic arch occlusion. Our findings uncover a previously unknown mechanism of oxidative stress in the pathogenesis of SCIR injury that promotes autophagic cell death and delineate a crucial protective function of H_2_S in SCIR.

## 2. Materials and Methods

### 2.1. Animals

Eight-week-old male Sprague-Dawley rats were purchased from the Shanghai Laboratorial Animal Center at the Chinese Academy of Sciences. The animals were housed with ad libitum access to water and food in an air-conditioned room with a 12 h light-dark cycle, at 21°C to 23°C and 60% relative humidity, in the animal facility at Ruijin Hospital, Shanghai Jiao Tong University School of Medicine, China.

### 2.2. Ethics Statement

All animal experiments were performed in accordance with the protocol approved by the Shanghai Jiao Tong University (SJTU) Animal Care and Use Committee [IACUC protocol number: SYXK (Shanghai) 2011-0113] and in accordance with the Ministry of Science and Technology of the People's Republic of China Animal Care guidelines. All surgeries were performed under anesthesia, and all efforts were made to minimize suffering.

### 2.3. Spinal Cord Ischemic Reperfusion (SCIR) Injury Model

The ischemic reperfusion (I/R) model was generated using a modification of a method reported before [1]. Briefly, all rats were neurologically intact before the experiment and anesthetized with 2.5% sodium-pentobarbital (60 mg/kg) injected intraperitoneally. In the rats of the I/R group, the abdominal aorta was blocked above the right renal artery near the heart using a 50 g aneurysm clip for 60 min. The rats in the sham group underwent the same procedure, but no occlusion of the aorta was performed. All rats were placed in a box at 28°C to recover from anesthesia and were subsequently placed in separated cages with ad libitum access to food and water.

### 2.4. Drug Preparation

The following reagents were purchased from Sigma-Aldrich: 3-methyladenine (3-MA, M9281), N-acetyl-L-cysteine (NAC, A7250), sodium hydrosulfide (NaHS · H2O, 13590), Ly294002 (L9908), and rapamycin (R0395). 3-MA, Ly294002, and rapamycin were dissolved in dimethyl sulfoxide to yield a stock solution of 25 mg/mL and further diluted in phosphate-buffered saline (PBS) for the final dose before intraperitoneal injection. NAC and NaHS were dissolved in PBS before intraperitoneal injection.

### 2.5. SCIR Treated with Different Drugs

The sham group (*n* = 6) underwent the surgical procedure without aortic clipping. The I/R group (*n* = 6) received abdominal aortic exposure and cross-clamping for 60 min followed by intraperitoneal injection of an equivalent volume of 0.9% saline solution immediately after reperfusion. The rats in the I/R + 3-MA group (*n* = 6) and I/R + NAC group (*n* = 6) also received the same surgical procedure as the I/R group, but were treated with 3-MA (2.5 mg/kg) [23] or NAC (300 mg/kg) [24] immediately after I/R injury, respectively. Rats in the I/R + NaHS group (*n* = 6) were intraperitoneally injected with NaHS · H2O (5.6 mg/kg) [25], 1 h before the onset of spinal cord I/R. Rats in the NaHS + I/R + Ly294002 group (*n* = 6) were intraperitoneally injected with Ly294002 (1.5 mg/kg) [26], 0.5 h before the administration of NaHS and subsequent spinal cord I/R. Rats in the I/R + NaHS + rapamycin group (*n* = 6) were intraperitoneally injected with rapamycin (0.5 mg/kg) [27], 0.5 h before the administration of NaHS and subsequent SCIR. All experiments were repeated three times.

### 2.6. Neurological Function Assessment

Locomotor recovery after SCIR was assessed using the Basso, Beattie, and Bresnahan (BBB) open-field locomotor scale [28] ranging from 0 (complete paralysis) to 21 (normal locomotion). The BBB scores were recorded at 1, 6, 12, and 24 h in the acute phase after reperfusion by two experienced investigators who were blind to the whole experiment. Disagreements were solved through discussion to reach a consensus.

### 2.7. Immunofluorescence Staining of Bax, Caspase-3, and LC3

The frozen sections were washed with PBS for 10 min, followed by washing with PBS containing 0.1% Tween for 10 min, and then blocked with 5% bovine serum albumin (BSA, Sigma) for 30 min at room temperature. The sections were incubated in permeabilization solution (1% Triton X-100) for 15 min at room temperature and then incubated in primary rabbit anti-Bax antibody (1 : 100; Santa Cruz Biotechnology) or primary rabbit anti-Caspase 3 antibody (1 : 100; Santa Cruz Biotechnology) or primary rabbit anti-LC3B antibody (1 : 200; Cell Signaling Technology) diluted in PBS overnight at 4°C. After rinsing with PBST, the sections were incubated with goat anti-rabbit Ig (immunoglobulin) G Alexa Fluor 488 secondary antibody (1 : 500; Molecular Probes) for 1 h at room temperature. The sections were mounted with ProLong® Gold antifade reagent with DAPI to label the nuclei (Molecular Probes).

### 2.8. Terminal Deoxynucleotidyl Transferase-Mediated dUTP Nick End Labeling (TUNEL) Assay

In order to identify DNA fragmentation, TUNEL assay was performed. Apoptotic cells in the frozen spinal cord sections were stained using an in situ cell death detection kit, TMR red (Roche Diagnostics), according to the manufacturer's instructions. Briefly, sections were washed in PBS and incubated in permeabilization solution for 15 min at room temperature and then in the TUNEL solution containing TMR-dUTP for 1 h at 37°C. After labeling, cell nuclei were labeled with ProLong Gold antifade reagent with DAPI (Molecular Probes).

### 2.9. Immunohistochemical Staining of Bax

The paraffin sections were deparaffinized and rehydrated. Antigen retrieval was performed in accordance with the manufacturer's instructions for the citrate antigen retrieval solution (Beyotime, China). The sections were incubated in hydrogen peroxide to quench endogenous peroxidases and then blocked with 5% BSA (Sigma) for 30 min at room temperature. The sections were incubated in primary rabbit anti-Bax antibody (1 : 100; Abcam) diluted in PBS overnight at 4°C. The Vectastain Elite ABC Kit (Vector Laboratories, USA) was used according to the manufacturer's instructions. Positive staining was visualized with diaminobenzidine (DAB; ImmPACT DAB, Vector Laboratories, USA). The sections were counterstained with hematoxylin for 10 s and dipped in acid alcohol as needed before being dehydrated and coverslipped.

### 2.10. Western Blotting

The spinal cords were homogenized in a radioimmunoprecipitation assay (RIPA) buffer (Beyotime, Nanjing, China) with phenylmethanesulfonyl fluoride (PMSF) protease and a phosphatase inhibitor cocktail (CWBIO, Shanghai, China). The homogenates were clarified using centrifugation at 12000*g* for 15 min at 4°C. The concentration of protein samples was determined using the BCA protein assay kit (Beyotime, China). Aliquots of protein (50 *μ*g/lane) were fractionated using 10% sodium dodecyl sulfate polyacrylamide gel electrophoresis (SDS-PAGE). After electrophoresis, the proteins on the gel were electroblotted onto polyvinylidene difluoride membranes (0.45 *μ*m, Millipore, USA). The membranes were blocked in Tris-buffered saline/Tween (20 mmol/L Tris, pH 7.5, 0.5 mol/L NaCl, and 0.1% Tween 20) containing 5% nonfat dry milk for 1 h at room temperature and subsequently incubated with primary antibody overnight at 4°C. The membranes were incubated with secondary antibody for 90 min at room temperature. The chemiluminescence results were recorded using an imaging system (ImageQuant LAS 4000 mini, General Electric, USA). Signal intensities were quantified using Image-Pro Plus software. The antibodies used were as follows: rabbit anti-mTOR (1 : 1000; Cell Signaling Technology), rabbit anti-p-mTOR (1 : 1000; Cell Signaling Technology), rabbit anti-AKT (1 : 1000; Cell Signaling Technology), rabbit anti-p-AKT (1 : 1000; Cell Signaling Technology), rabbit anti-SQSTM1/p62 (1 : 1000; Abcam), rabbit anti-Beclin 1 (1 : 1000; Cell Signaling Technology), rabbit anti-Atg12 (1 : 1000; Cell Signaling Technology), rabbit anti-cleaved Caspase 3 (1 : 1000; Cell Signaling Technology), rabbit anti-BCL2 (1 : 1000, Abcam), rabbit anti-Bax (1 : 1000, Cell Signaling Technology), rabbit anti-LC3B (1 : 1000, Cell Signaling Technology), mouse anti-*β*-Actin (1 : 1000, Cell Signaling Technology), and horseradish peroxidase- (HRP-) conjugated secondary antibody (1 : 5000, Jacksion). The intensities of the protein bands were quantified by densitometry analysis using NIH Image J software.

### 2.11. Oxidative Stress Assay

Superoxide dismutase (SOD) activity and malondialdehyde (MDA) concentration in the spinal cord tissue were measured using an oxidative stress assay. Fresh spine cord tissue was taken and washed with precooled PBS. It was converted to 100 g/L of spine cord homogenates in a homogenizer filled with 9 times the mass of precooled PBS. The homogenates were centrifuged at low temperature for 15 min at a speed of 3500 r/min. Proper amount of supernatant was given to perform tissue protein quantification. Levels of SOD and MDA were determined in accordance with the specifications of the SOD kit (Dojindo Molecular Technologies, Japan) and the MDA kit (Nanjing Jiancheng Bioengineering Institute, China). The protein concentration of the samples was determined using a BCA protein assay kit.

### 2.12. Statistical Analysis

All data are present as mean ± SEM. We used two-tailed *t*-tests to determine significances between two groups. We did analyses of multiple groups by one-way or two-way ANOVA with Bonferroni posttest of GraphPad prism version 5. For all statistical tests, we considered *P* value < 0.05 to be statistically significant.

## 3. Results

### 3.1. Both Apoptosis and Autophagy Increase in Neuronal Cells after SCIR Injury

**Figure 1:**
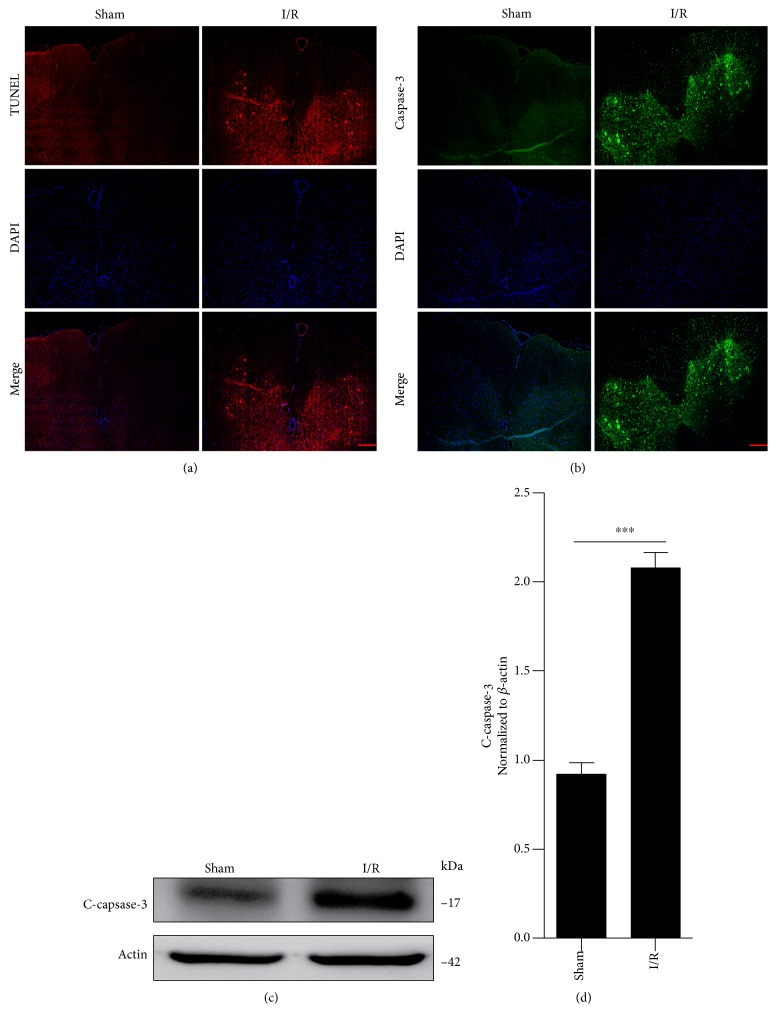
SCIR injury induces neuronal cell apoptosis. Immunofluorescence analysis of TUNEL (a) and caspase-3 (b) in the spinal cord after I/R. This image shows results obtained from six rats with I/R. Scale bars represent 10 *μ*m. (c) Western blot of cleaved caspase-3 in the spinal cord extracts from normal and I/R rats. (d) Densitometric analysis of the immunoblot reported in Figure 1(c). Samples from six normal and six I/R rats were pooled together. ^∗∗∗^*P* < 0.001. Data were analyzed using *t*-test and represent three independent experiments. SCIR: spinal cord ischemia reperfusion; TUNEL: terminal deoxynucleotidyl transferase-mediated dUTP nick end labeling; I/R: ischemia reperfusion.

In order to investigate the causal relationship between autophagy and apoptosis, we first detected levels of apoptosis after SCIR injury. The immunofluorescence staining results of the TUNEL showed that SCIR injury increased spinal cord neuronal cell death (Figure 1(a)). Furthermore, the immunofluorescence (Figure 1(b)) and Western blotting (Figures 1(c) and 1(d)) for caspase-3, which is best known for its role in the execution phase of apoptosis [29], also revealed that SCIR injury induced significant levels of apoptosis in neuronal cells.

**Figure 2:**
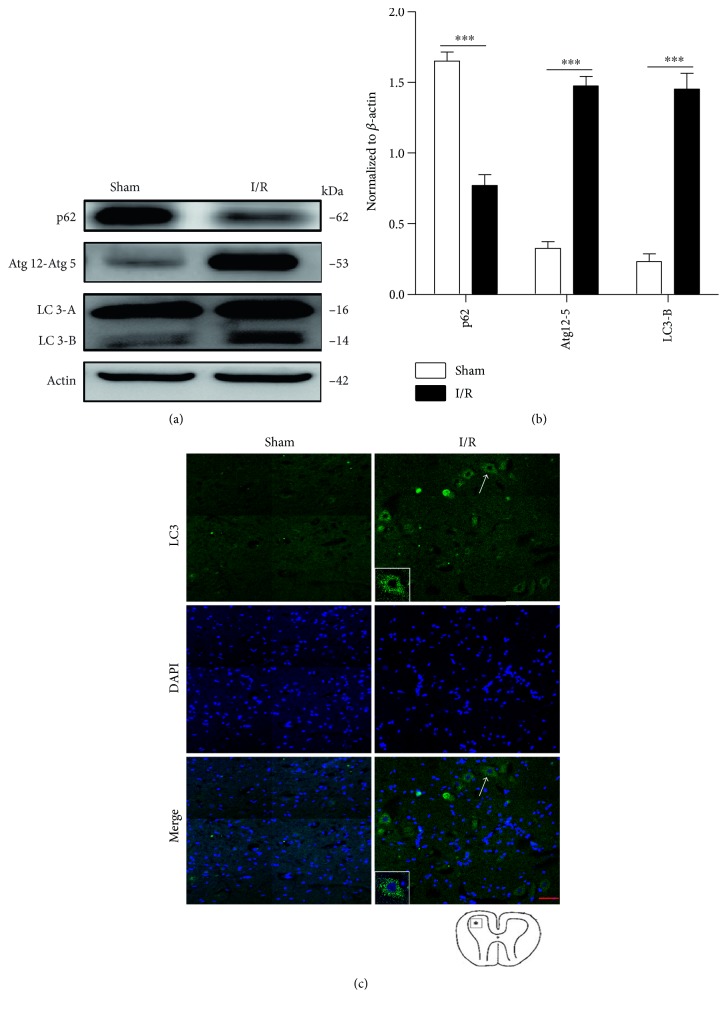
SCIR injury induces neuronal cell autophagy. (a) Western blots of p62, Atg12-Atg5, and LC3 in the spinal cord extracts from normal and I/R rats. Samples from six normal and six I/R rats were pooled together. (b) Densitometric analysis of the immunoblot reported in Figure 2(a). (c) Immunofluorescence analysis of LC3 in the spinal cord after I/R. This image represents six rats with I/R. Scale bar represent 10 *μ*m. Arrows designate regions of 400x magnification shown in insets. ^∗∗∗^*P* < 0.001. Data were analyzed using one-way ANOVA. Data represent three independent experiments. SCIR: spinal cord ischemia reperfusion; I/R: ischemia reperfusion; LC3: microtubule-associated protein 1 light chain 3.

Further, we detected the induction of autophagy in the pathogenesis of SCIR injury. The conversion of LC3-I to LC3-II is essential for autophagosome formation, and it is considered an indicator of autophagy induction [30]; the Western blot results showed that SCIR injury increased the accumulation of LC3-II in neuronal cells compared with the sham group (Figure 2(a)). Recently, Atg5, derived from preautophagosomal structures, has been shown to play an important role in the elongation of phagophores [31]; we observed that Atg5 levels increased significantly after SCIR injury (Figure 2(a)). In addition, levels of p62, another autophagy marker, which works as the LC3-II-binding protein that ties ubiquitinated protein aggregates to the autophagosome, were found to be decreased after SCIR injury (Figures 2(a) and 2(b)). Induction of autophagy was also confirmed by immunofluorescence analysis. The results showed that autophagosomes and autolysosomes were rarely detected in normal controls, whereas their levels were increased significantly after I/R, as demonstrated by increased punctate fluorescence staining signals of LC3 in the cytoplasm of neuronal cells (Figure 2(c)). Taken together, our findings demonstrate that both autophagy and apoptosis were increased in neuronal cells after SCIR injury.

### 3.2. Autophagy Promotes Neuronal Cell Death in SCIR Injury

**Figure 3:**
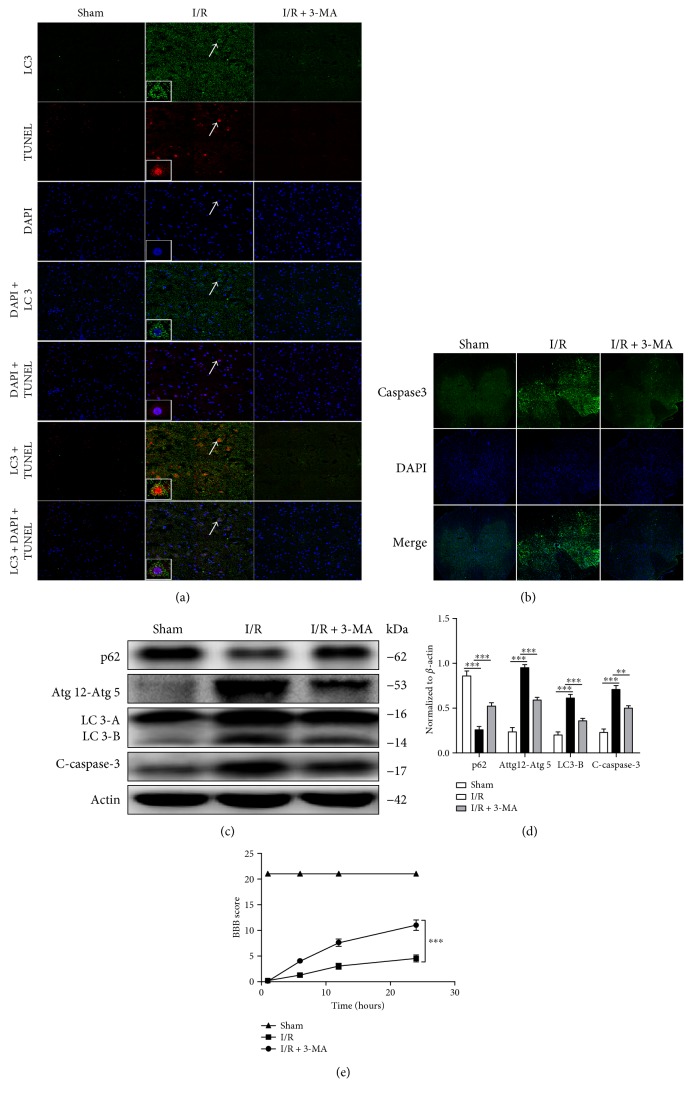
Autophagy promotes neuronal cell apoptosis in SCIR injury. (a) Immunofluorescence analysis of TUNEL and LC3 in the spinal cord after I/R treated with or without 3-MA. (b) Immunofluorescence analysis of caspase-3 in the spinal cord after I/R treated with or without 3-MA. (c) Western blot analysis of p62, Atg12-5, LC3-II, and cleaved caspase-3 after I/R injury treated with or without 3-MA. (d) Densitometric analysis of the immunoblot reported in Figure 3(c). (e) BBB scores of animals after SCIR treated with or without 3-MA. Images represent six rats with I/R treated with or without 3-MA. Scale bars represent 10 *μ*m. ^∗∗∗^*P* < 0.001. Data were analyzed using one-way ANOVA in (d) and two-way ANOVA in (e) and represent three independent experiments. SCIR: spinal cord ischemia reperfusion; TUNEL: terminal deoxynucleotidyl transferase-mediated dUTP nick end labeling; LC3: microtubule-associated protein 1 light chain 3; I/R: ischemia reperfusion; 3-MA: 3-methyladenine; BBB: Basso, Beattie, and Bresnahan; ANOVA: analysis of variance.

We observed that both autophagy and apoptosis were increased in neuronal cells after SCIR injury. Previous work has shown that autophagy might have a detrimental effect on the pathology of cardiac ischemia reperfusion [32]. We therefore hypothesized that autophagy might promote neuronal cell apoptosis in the pathogenesis of SCIR injury. In order to test our hypothesis, we first performed loss-of-function experiments, in which we inhibited autophagy by 3-methyladenine (3-MA). The results showed that with decreased autophagosome formation, neuronal cell death was significantly reduced in the 3-MA-treated group (Figure 3(a)), as the immunofluorescence analysis revealed that TUNEL-positive neuronal cells decreased significantly in the 3-MA group compared with the control group. Interestingly, we found that TUNEL-positive cell nuclei were surrounded by LC3, which further suggests that autophagic neuronal cell death was induced in the spinal cord in response to I/R. Consistent with TUNEL staining, the results showed that apoptosis in neuronal cells induced by I/R was reduced after autophagy was inhibited by 3-MA (Figure 3(b)). In addition, a decrease in neuronal cell apoptosis and autophagy triggered by 3-MA was further shown by the Western blotting (Figures 3(c) and 3(d)). Finally, the BBB scores also confirmed the detrimental effect of autophagy on neuronal cell function, as decreased BBB scores were restored much quicker after autophagy was blocked in SCIR injury (Figure 3(e)). This indicates that the deteriorated neurological and motor functions were restored by 3-MA in SCIR injury. Taken together, our findings indicate that autophagy promotes neuronal cell death in SCIR injury.

### 3.3. Oxidative Stress Induces Neuronal Autophagic Cell Death in SCIR Injury

**Figure 4:**
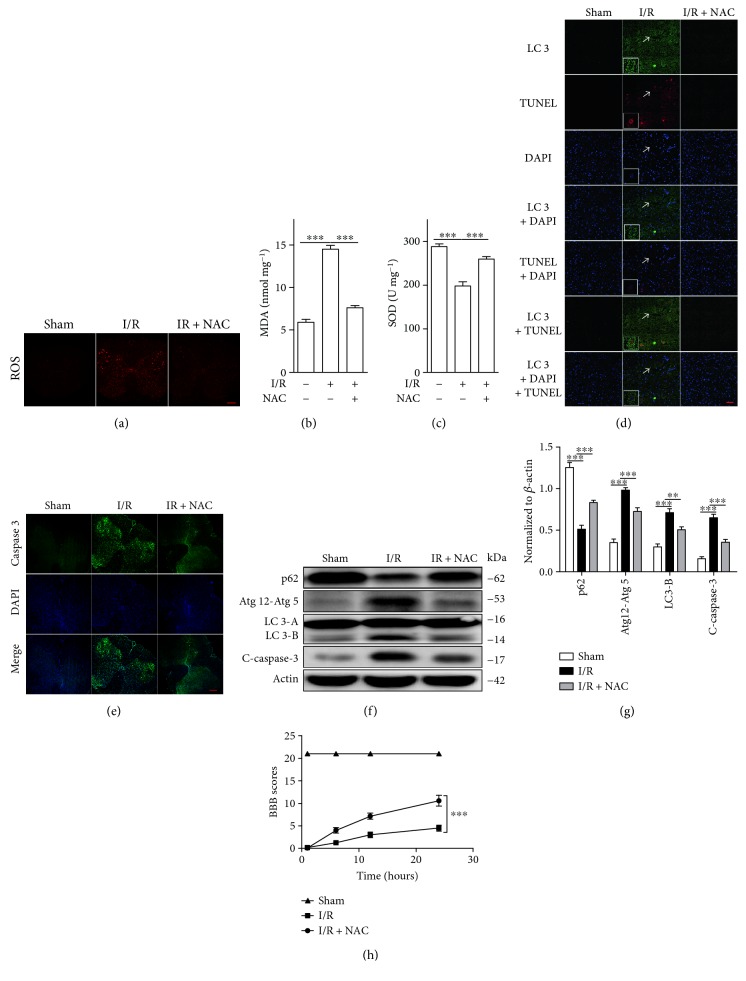
Oxidative stress induces neuronal autophagic cell death in SCIR injury. (a) Immunofluorescence analysis of ROS in the spinal cord after SCIR treated with or without NAC. (b) MDA concentration and (c) SOD activity in the spinal cord after I/R treated with or without NAC. (d) Immunofluorescence analysis of TUNEL and LC3 in the spinal cord after I/R treated with or without NAC. (e) Immunofluorescence analysis of caspase-3 in the spinal cord after I/R treated with or without NAC. (f) Western blot analysis of p62, Atg12-5, LC3-II, and cleaved caspase-3 after I/R injury treated with or without NAC. (g) Densitometric analysis of the immunoblot reported in Figure 4(f). (h) BBB scores of animals after SCIR treated with or without NAC. Images represent six rats with I/R treated with or without NAC. Scale bars represent 10 *μ*m. ^∗∗∗^*P* < 0.001. Data were analyzed using one-way ANOVA in (b), (c), and (g) and two-way ANOVA in (h). Data represent three independent experiments. SCIR: spinal cord ischemia reperfusion; ROS: reactive oxygen species; NAC: N-acetyl-L-cysteine; MDA: malondialdehyde; SOD: superoxide dismutase; I/R: ischemia reperfusion; TUNEL: terminal deoxynucleotidyl transferase-mediated dUTP nick end labeling; BBB: Basso, Beattie, and Bresnahan; ANOVA: analysis of variance.

Having identified that autophagy plays a detrimental role in neuronal cell death, we further sought to explore the mechanisms involved in the induction of autophagic cell death in SCIR injury. Previous studies have reported that ROS exert a crucial role in neuronal cell damage during SCIR injury [5]. Oxidative stress has also been reported to be associated with autophagy in the neonatal hypoxia-ischemia brain [33]. We therefore sought to determine whether oxidative stress was the trigger of autophagic death in neuronal cells during SCIR injury. First, we measured levels of oxidative stress in SCIR injury. Immunofluorescence analysis results showed that I/R injury led to increased ROS expression in neuronal cells of the spinal cord (Figure 4(a)). Malondialdehyde (MDA) is a lipid peroxidation marker, while superoxide dismutase (SOD) is an antioxidant enzyme that protects cells against oxidative injury by scavenging superoxide anions. The concentration of MDA and enzymatic activities of SOD are often used to evaluate oxidative and antioxidative reactions [34]. Our results showed that, accompanied by an increase in the concentration of MDA (Figure 4(b)), the activity of SOD was significantly reduced in the SCIR injury group compared with the control samples (Figure 4(c)). Taken together, our findings indicate that oxidative stress is upregulated during SCIR injury.

In order to further delineate the function of oxidative stress in neuronal autophagic cell apoptosis, we next attempted to reduce oxidative stress in SCIR injury. N-acetylcysteine (NAC) is a widely used antioxidant drug whose metabolism results in an increase in intracellular glutathione that produces sulfhydryl groups directly eliminating ROS [35, 36]. Our results showed that in contrast with the decrease in ROS expression and the reduction in MDA concentration, the decreased activity of SOD was restored by NAC in SCIR injury (Figures 1(a), 1(b), and 1(c)). This indicates that NAC successfully reduced oxidative stress in SCIR injury. Furthermore, consistent with the reduction in oxidative stress, we observed lower levels of autophagic neuronal cell death, detected by TUNEL and LC3 double staining (Figure 4(d)). Accordingly, the immunofluorescence results of caspase-3 further confirmed that neuronal cell apoptosis during SCIR injury was triggered by oxidative stress (Figure 4(e)). Furthermore, the Western blot results confirmed the inhibiting effect of NAC on neuronal cell apoptosis and autophagy (Figures 4(f) and 4(g)). Finally, the BBB scores further confirmed that oxidative stress exerts a detrimental effect on neurological and motor function (Figure 4(h)). Taken together, our findings indicate that oxidative stress induces neuronal autophagic cell death in SCIR injury.

### 3.4. H_2_S Protects Neuronal Cells from Autophagic Death by Reducing Oxidative Stress

**Figure 5:**
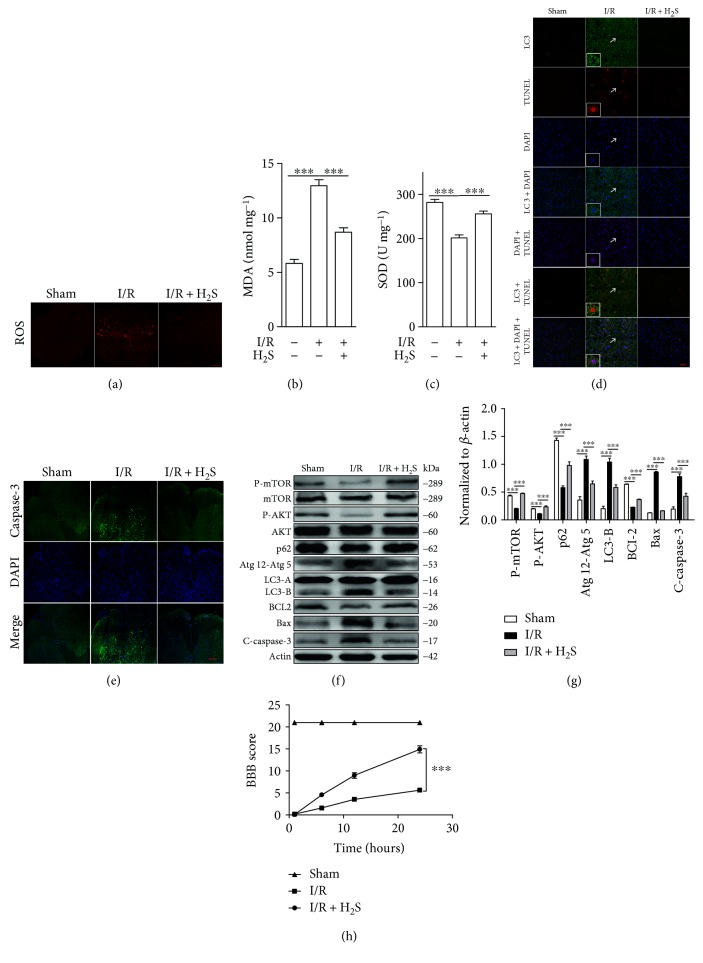
H_2_S inhibits neuronal autophagic cell death after SCIR injury. (a) Immunofluorescence analysis of ROS in the spinal cord after I/R treated with or without H_2_S. (b) MDA concentration and (c) SOD activity in the spinal cord after I/R treated with or without H_2_S. (d) Immunofluorescence analysis of TUNEL and LC3 in the spinal cord after I/R treated with or without H_2_S. (e) Immunofluorescence analysis of caspase-3 in the spinal cord after I/R treated with or without H_2_S. (f) Western blot analysis of cleaved caspase-3, Bax, BLC2, LC3, Atg12-Atg5, p62, p-AKT, and p-mTOR in the spinal cord extracts from normal and I/R rats treated with or without H_2_S. (g) Densitometric analysis of the immunoblot reported in (f). (h) BBB scores of animals after SCIR treated with or without H_2_S. Images represent six rats with I/R treated with or without H_2_S. Scale bars represent 10 *μ*m. ^∗∗∗^*P* < 0.001. Data were analyzed using one-way ANOVA in (b), (c), and (g) and two-way ANOVA in (h) and represent three independent experiments. H_2_S: hydrogen sulfide; SCIR: spinal cord ischemia reperfusion; ROS: reactive oxygen species; I/R: ischemia reperfusion; MDA: malondialdehyde; SOD: superoxide dismutase; TUNEL: terminal deoxynucleotidyl transferase-mediated dUTP nick end labeling; LC3: microtubule-associated protein 1 light chain 3; BBB: Basso, Beattie, and Bresnahan; ANOVA: analysis of variance.

Having observed oxidative stress-induced neuronal autophagic cell death during SCIR injury, we next sought to alleviate SCIR injury by targeting oxidative stress. H_2_S is an endogenously generated gaseous signaling molecule, which has been shown to exert cardioprotective effects by neutralizing ROS in myocardium I/R injury [19–21]. We therefore hypothesized that H_2_S might protect neuronal cells from SCIR injury by reducing oxidative stress. In order to test our hypothesis, we first detected levels of oxidative stress in SCIR after H_2_S administration. Immunofluorescence results showed that the upregulation of ROS in the spinal cord was significantly inhibited by H_2_S (Figure 5(a)). Accordingly, we further observed that H_2_S significantly restrained SCIR injury-induced MDA (Figure 5(b)), whereas the decreased activity of SOD was largely restored (Figure 5(c)). These results demonstrate that H_2_S successfully reduces oxidative stress in SCIR injury.

Accompanied by a reduction in oxidative stress, TUNEL-LC3 double staining results suggested that autophagic neuronal cell death is also significantly inhibited by H_2_S in SCIR injury (Figure 5(d)) and that this reduction in cell death includes apoptosis (Figure 5(e)). Furthermore, the inhibition of neuronal autophagic cell death by H_2_S was also confirmed through Western blotting, as levels of cleaved caspase-3, Bax, LC3-II, and Atg5 were all largely decreased after H_2_S administration in SCIR injury, whereas p62 expression was increased (Figures 5(f) and 5(g)). Furthermore, the protective role of H_2_S in neurological and motor function was also shown by the BBB score (Figure 5(h)). Taken together, our findings indicate that H_2_S protects neuronal cells from autophagic death.

### 3.5. H_2_S Protects Neuronal Cells from Autophagic Death through the AKT-mTOR Pathway

**Figure 6:**
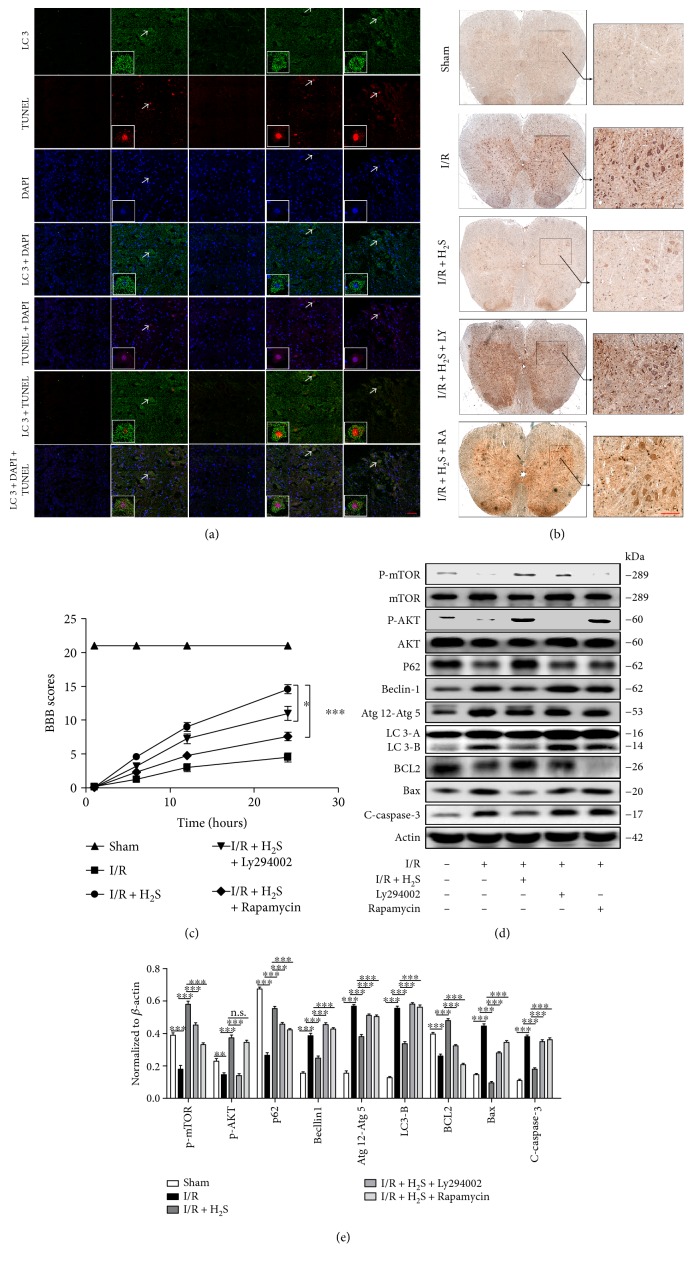
H_2_S inhibits neuronal autophagic cell death via the AKT-mTOR pathway. (a) Immunofluorescence analysis of TUNEL and LC3 in the spinal cord after I/R treated with or without Ly294002 or rapamycin. (b) Immunohistochemistry staining of Bax in the spinal cord after I/R treated with or without Ly294002 or rapamycin. (c) BBB scores of animals after SCIR treated with or without Ly284002 or rapamycin. (d) Western blots of cleaved caspase-3, Bax, BCL2, LC3, Atg12-Atg5, p62, P-AKT, and P-mTOR in spinal cord extracts from normal and I/R rats treated with or without H_2_S. (e) Densitometric analysis of the immunoblot reported in (d). Samples from six normal and six I/R rats were pooled together. Images represent six rats per group with different treatments. Scale bar represent 10 *μ*m. ^∗^*P* < 0.05, ^∗∗^*P* < 0.01, ^∗∗∗^*P* < 0.001. Data were analyzed using one-way ANOVA in (e) and two-way ANOVA in (c). H_2_S: hydrogen sulfide; LY: Ly294002; RA: rapamycin; mTOR: the mammalian target of rapamycin; LC3: microtubule-associated protein 1 light chain 3; I/R: ischemia reperfusion; TUNEL: terminal deoxynucleotidyl transferase-mediated dUTP nick end labeling; BBB: Basso, Beattie, and Bresnahan; SCIR: spinal cord ischemia reperfusion; ANOVA: analysis of variance.

After having delineated a key role of H_2_S in inhibiting neuronal autophagic cell death, we next sought to explore the intrinsic mechanisms by which H_2_S exerts these protective effects. AKT and mTOR pathways have been shown to be involved in the inhibition of autophagy in neonatal rat cardiomyocytes exposed to hypoxia/reoxygenation by H_2_S. Blocking AKT by Ly294002 (an AKT inhibitor) or inactivating mTOR by rapamycin increased autophagy and attenuated the antiautophagy effect of H_2_S [4, 22]. In addition, our results also indicated that SCIR injury significantly inhibited AKT and mTOR phosphorylation, whereas H_2_S largely restored levels of AKT and mTOR (Figure 5(f)). Therefore, we hypothesized that H_2_S inhibited neuronal autophagic death via AKT and mTOR. In order to test our hypothesis, we first blocked AKT with Ly294002 and mTOR with rapamycin, respectively. The double staining of TUNEL and LC3 in the spinal cord revealed that H_2_S reduced autophagic neuronal cell death after I/R injury, whereas this process was significantly inhibited by Ly294002 or rapamycin (Figure 6(a)). This implies that the activation of both the AKT and the mTOR pathways is essential for the protective role of H_2_S in preventing neuronal autophagic death. Furthermore, the role of H_2_S in inhibiting SCIR injury-induced neuronal death via AKT and mTOR was also confirmed by the Bax immunohistochemistry staining (Figure 6(b)). Consistent with our findings regarding autophagic cell death, the BBB scores further highlighted the critical role of the activation of both the AKT and the mTOR pathways in the H_2_S-related restoration of neurological and motor function after SCIR injury (Figure 6(c)). Finally, the Western blot results showed that in addition to the reduced phosphorylation of AKT and mTOR, Ly294002 or rapamycin administration significantly increased levels of cleaved caspase-3, Bax, LC3-Π, Atg5, and p62 in SCIR injury, which further demonstrated that AKT and mTOR pathway activation is essential for H_2_S-inhibited neuronal autophagic cell death (Figures 6(d) and 6(e)). Interestingly, we observed that apart from blocking H_2_S-induced AKT phosphorylation, the AKT inhibitor Ly294002 also significantly inhibited mTOR activation; however, the mTOR inhibitor rapamycin had no effect on H_2_S-induced AKT phosphorylation (Figures 6(d) and 6(e)), which suggests that mTOR works as the downstream molecule of AKT in H_2_S-protected neuronal autophagic death. Taken together, our findings indicate that H_2_S inhibits neuronal autophagic cell death through the AKT-mTOR pathway.

## 4. Discussion

Autophagy plays an important role in the degradation of cytoplasmic constituents in the autophagy-lysosomal pathway [37]. Increasing studies have reported that levels of autophagy are elevated in the central nervous system under stress conditions and that this process plays an important role in spinal cord injury [38–41]. However, the mechanisms underlying autophagy after SCIR injury are largely unknown. Furthermore, autophagy seems like a double-edged sword, and whether it plays a protective or a detrimental role in SCIR injury remains controversial [2]. Here, we observed that both apoptosis and autophagy increased in neuronal cells after SCIR injury. Furthermore, our results revealed that autophagy promoted autophagic cell death after SCIR injury, as we found that a significant number of TUNEL-positive cells were colabeled with the autophagy marker LC3, and the autophagy inhibitor 3-MA led to a decrease in the number of TUNEL-positive cells, thereby ameliorating neurological and motor function. In addition, we showed that oxidative stress is the main trigger of autophagic neuronal cell death after SCIR injury, as NAC, accompanied by a reduction in MDA and increase in SOD, largely reduced autophagic cell death and alleviated neurological and motor function in SCIR injury. Finally, we identified H_2_S, which reduced autophagic cell death and restored neurological and motor function significantly by reducing oxidative stress in SCIR injury. Moreover, H_2_S inhibited autophagic neuronal cell death via the AKT-mTOR pathway. The identification of oxidative stress as a trigger of autophagic cell death in SCIR injury and the discovery of H_2_S exerting a protective role in SCIR injury provide a new mechanism for understanding innate immune responses during SCIR injury. Moreover, these findings also suggest potential applications of H_2_S in SCIR treatment.

The normal metabolic balance of eukaryotic cells is maintained by two main pathways: the ubiquitin-proteasome pathway and the autophagy-lysosomal pathway [15]. As an intracellular catabolic mechanism, autophagy maintains a balance between protein synthesis and degradation and can play a cell-protective or cell-destructive role, depending on the specific pathological events [2]. Previous studies suggest that autophagy contributes to cytoprotection in neurodegenerative disease and traumatic brain injury [42–45]. In contrast, recent reports indicate that the activation of autophagy induces cell death in a myocardial ischemia and reperfusion model. In addition, autophagy can lead to autophagic cell death in cerebral ischemia and in renal ischemia and reperfusion injury [8, 13]. Moreover, it has been found that autophagy plays opposing roles during the bimodal stage. Early activated autophagy (SCIR injury induced by a 14 min occlusion of the aortic arch) alleviates spinal cord I/R injury via inhibiting apoptosis and inflammation; later excessively elevated autophagy levels, however, aggravate I/R injury by inducing autophagic cell death [2]. Concurrent with these findings, we found that after 1 h of aortic arch occlusion, autophagy exerts a destructive role on neurons in SCIR injury. This was evident in a significant number of TUNEL-positive cells colabeled with the autophagy marker LC3, as well as in the finding that the autophagy inhibitor 3-MA led to a decrease in TUNEL-positive cells and restored neurological and motor function as indicated by BBB scores.

The cellular and molecular mechanisms that result in ischemia-reperfusion damage to the medulla spinalis have not been explained clearly. The rapid increase in free radicals and oxidative stress is currently considered the most critical event for irreversible cellular damage in SCIR injury [46]. Neurotoxicity, intracellular calcium increase, lipid peroxidation, and free radical formation are all part of a complex relationship in the pathophysiology of spinal cord ischemia. Reperfusion restores lost cellular functions during ischemia; however, it increases blood flow and tissue oxygenation and thereby causes further damage in ischemic tissues via the formation of reactive oxygen radicals [47]. Besides triggering cell damage, oxidative stress has also been shown to be a trigger of autophagy activation in neuronal cells [33]. Concurrent with this, we showed that the induction of autophagic cell death during SCIR injury was oxidative stress-dependent. We first found an increase in ROS expression and in MDA concentration in the spinal cord, while the activity of SOD was significantly reduced after I/R injury. Second, we observed that accompanied by a decrease in ROS/MDA and an increase in SOD, NAC inhibited autophagic cell death and restored neurological and motor function in SCIR injury. This reveals that oxidative stress plays a crucial role in nerve destruction after SCIR injury. Therefore, pharmacological therapies targeting oxidative stress may be critical for restraining SCIR injury.

Hydrogen sulfide (H_2_S) is a gaseous messenger molecule that has recently been implicated in various physiological-pathological processes in mammals, including vascular relaxation, angiogenesis, I/R injury of the heart, and the function of ion channels [19]. Recently, hydrogen sulfide has been shown to exhibit protective effects against secondary neuronal injury through the inhibition of malondialdehyde and to suppress the effects of various reactive oxygen species [48, 49]. Moreover, the administration of sodium hydrosulfide, a hydrogen sulfide donor, has been shown to reduce infarct volume and to improve neurological function by reducing apoptosis in cerebral I/R injury [50]. However, whether H_2_S could play a neuronal protective role in SCIR injury is still unclear. In the present study, we found that H_2_S (NaHS administration) attenuated the increase in MDA concentration, while SOD activity in the spinal cord after I/R injury increased. Furthermore, accompanied by reduced autophagic cell death, neuronal and motor functions were restored after SCIR injury. Moreover, our results revealed that H_2_S inhibited autophagic cell death via the AKT-mTOR pathway. This was evident in a reduction of H_2_S-induced AKT and mTOR phosphorylation, mediated by the AKT inhibitor Ly294002 and the mTOR inhibitor rapamycin, respectively, and in a decrease in autophagic cell death as well as the restoration of neuronal and motor function. Interestingly, accompanied by a decrease in AKT phosphorylation, we found that the AKT inhibitor Ly294002 also attenuated mTOR phosphorylation, while a mTOR inhibitor did not have the capacity to block AKT phosphorylation. This implies that mTOR is the downstream molecule of AKT in the neuronal protective pathway of H_2_S. The crucial protective roles of AKT and mTOR in SCIR injury are consistent with previous reports, as studies have demonstrated that PI3K/AKT and its downstream mTOR complexes are involved in the promotion of cell survival and growth in SCIR injury [51].

As the final metabolite of sulfur-containing amino acids, H_2_S exists both in the gaseous form and in the dissolved NaHS form in vivo. NaHS can be hydrolyzed into Na^+^ and HS^−^, the latter being able to bind H^+^ in the body to produce H_2_S, which forms a dynamic equilibrium with NaHS [25]. The H_2_S concentration in a NaHS solution is stable, and an NaHS solution has been widely used as an H_2_S donor [52]. Some studies have shown a protective effect of a low dose of H_2_S in an in vivo study [53]. In an in vitro study, a physiological concentration of H_2_S has been shown to protect neurons against hypoxic injury [54]. It has been reported that H_2_S levels above the physiological range can cause cytotoxicity to neurons [25]. In the present study, we injected NaSH · H2O intraperitoneally at a concentration of 5.6 mg/kg (approximately 57 *μ*mol/kg) [25] to examine the effect of exogenous H_2_S on SCIR injury. Although no obvious side effects of this concentration were identified in this study, a pretreatment with 180 *μ*mol/kg NaHS has been shown to aggravate neuronal injury after ischemia [25, 55]. It is possible that pretreatment with a lower dose of exogenous NaHS may increase and restore H_2_S levels to the physiological range and thus protect neurons from I/R injury. However, a higher dose of exogenous NaHS may contribute to levels of H_2_S above the physiological range and thus predisposes neurons to I/R injury. This may also partially explain the controversial findings regarding the effects of NaHS in different studies.

## 5. Conclusions

In conclusion, our study provides evidence that oxidative stress is the main trigger of autophagic cell death in SCIR injury and that H_2_S can exert a neuroprotective role in SCIR injury by reducing oxidative stress. Our findings uncover a new mechanism of SCIR injury and suggest potential applications of H_2_S in the treatment of SCIR injury.

## Abbreviations

SCIR: Spinal cord ischemia reperfusion injury
TUNEL: Terminal deoxynucleotidyl transferase-mediated dUTP-biotin nick end labeling assay
LC3: Microtubule-associated protein 1 light chain 3
3-MA: 3-Methyladenine
SOD: Superoxide dismutase
MDA: Malondialdehyde
NAC: N-acetylcysteine
H_2_S: Hydrogen sulfide
ROS: Reactive oxidative species
BBB score: Basso, Beattie, and Bresnahan score
C-caspase-3: Cleaved caspase-3
LY: Ly294002
RA: Rapamycin
I/R: Ischemia reperfusion injury
mTOR: The mammalian target of rapamycin.
